# Transcription Factor Pit-1 Affects Transcriptional Timing in the Dual-Promoter Human Prolactin Gene

**DOI:** 10.1210/endocr/bqaa249

**Published:** 2021-01-03

**Authors:** Anne V McNamara, Raheela Awais, Hiroshi Momiji, Lee Dunham, Karen Featherstone, Claire V Harper, Antony A Adamson, Sabrina Semprini, Nicholas A Jones, David G Spiller, John J Mullins, Bärbel F Finkenstädt, David Rand, Michael R H White, Julian R E Davis

**Affiliations:** 1 Systems Microscopy Centre, Division of Molecular and Cellular Function, School of Biological Sciences, Faculty Biology, Medicine & Health, Manchester Academic Health Sciences Centre, University of Manchester, Manchester, UK; 2 School of Life Sciences, University of Liverpool, Liverpool, UK; 3 Mathematics Institute & Zeeman Institute for Systems Biology, and Infectious Epidemiology Research, University of Warwick, Senate House Coventry, UK; 4 Division of Diabetes, Endocrinology & Gastroenterology, School of Medical Sciences, Faculty of Biology, Medicine & Health, Manchester Academic Health Sciences Centre, University of Manchester, Manchester, UK; 5 Department of Biology, Edge Hill University, Ormskirk, Lancashire, UK; 6 Genome Editing Unit, Faculty of Biology, Medicine & Health, Manchester Academic Health Sciences Centre, University of Manchester, Manchester, UK; 7 University/BHF Centre for Cardiovascular Science, The Queen’s Medical Research Institute, University of Edinburgh, Edinburgh, UK

**Keywords:** gene transcription, prolactin gene, pituitary, Pit-1, transcription timing, promoter

## Abstract

Gene transcription occurs in short bursts interspersed with silent periods, and these kinetics can be altered by promoter structure. The effect of alternate promoter architecture on transcription bursting is not known. We studied the human prolactin (hPRL) gene that contains 2 promoters, a pituitary-specific promoter that requires the transcription factor Pit-1 and displays dramatic transcriptional bursting activity and an alternate upstream promoter that is active in nonpituitary tissues. We studied large hPRL genomic fragments with luciferase reporters, and used bacterial artificial chromosome recombineering to manipulate critical promoter regions. Stochastic switch mathematical modelling of single-cell time-lapse luminescence image data revealed that the Pit-1–dependent promoter showed longer, higher-amplitude transcriptional bursts. Knockdown studies confirmed that the presence of Pit-1 stabilized and prolonged periods of active transcription. Pit-1 therefore plays an active role in establishing the timing of transcription cycles, in addition to its cell-specific functions.

Prolactin is a multifunctional mammalian hormone with a major role in lactation as well as other biological functions, including reproduction, immunomodulation and behavior (1,2). This polypeptide hormone mainly originates in the lactotroph cells of the anterior pituitary gland but is also expressed at extra-pituitary sites, such as brain decidualized endometrium, myometrium, and circulating lymphocytes, and hence requires tissue-specific transcriptional control mechanisms to regulate its functional versatility (1-3).

The human prolactin (hPRL) gene is located on the short arm of chromosome 6 and consists of 5 coding exons within a region of 10 kb. The gene has 2 distinct, alternative promoter elements that are 5.8 kb apart and show selective and tissue-specific activation (2,4). The proximal promoter is located immediately upstream of exon 1b and regulates hPRL transcription in the pituitary gland. This is often referred to as the pituitary-specific promoter and contains multiple binding sites for the pituitary-specific transcription factor Pit-1 (5,6). In lymphocytes and human endometrial cells, hPRL expression has been shown to be driven by an alternative promoter, upstream of the pituitary transcriptional start site (7). This evolved from a long terminal repeat-like transposon, resulting in transcription of the additional exon 1a, giving rise to messenger RNA (mRNA) that is 150 bp longer than the pituitary mRNA (2,8). The different sized transcripts do not give rise to different protein isoforms but are believed to contribute an additional level of regulation of prolactin (PRL) transcription in different cellular and functional contexts (pituitary *vs* extra-pituitary) by influencing the stability or translational efficiency of alternatively transcribed messages (2,8). Expression of the longer alternative promoter transcript has classically been viewed as a product of extra-pituitary sites and are considered to be Pit-1 independent (4,9,10).

Pit-1 (also known as POU1F1) is a member of the POU family of transcription factors that are characterized by the presence of a bipartite DNA binding domain, known as the POU domain (11). It is essential for the differentiation of lactotroph, somatotroph, and thyrotroph cells; Snell and Jackson dwarf mouse models carry mutations in the Pit-1 gene and show no development of these pituitary cell types (6), and Pit-1 mutations commonly result in hypopituitarism (12). Additionally, Pit-1 binding has been shown to play a crucial role in both basal and hormonally induced activity of the hPRL promoter (13-15).

The hPRL genomic locus has many conserved regions far upstream of the transcriptional start site. Outside of the genomic locus, the PRL gene is surrounded by over 1 Mb of noncoding DNA, a gene desert that could have a functional impact on hPRL regulation (16). It has therefore been important to develop strategies for studying the transcription control function of the complete genomic region. bacterial artificial chromosome (BAC)-based reporter systems permit the inclusion of far-distant regulatory elements and can prove particularly beneficial in analyzing function of promoters that comprise a complex array of cis-acting regulatory elements (7,17,18). We previously generated a BAC reporter construct that spanned 163 Kb of the hPRL genomic locus including 115 kb upstream and 38 kb downstream of the PRL gene and expressed firefly luciferase (Luc) under the control of the entire hPRL gene [referred to as hPRL wild-type (WT) BAC] (18).

In our earlier work, single-cell imaging of the hPRL WT BAC in pituitary cell lines and tissues showed that the hPRL gene displays dramatic pulses in transcriptional activity (19,20). This activity has been observed for many other genes, including the human growth hormone gene (21), and appears to be a general phenomenon that is intrinsic to gene regulation (21-27). The characteristics of these transcriptional pulses may be susceptible to modulation, as part of normal physiological control, and this might be expected to impact on overall levels of gene expression. In the present study, to study the role of Pit-1, we have compared the hPRL WT BAC with a construct in which the entire 5 kb pituitary promoter was deleted, leaving intact exon 1a and the upstream promoter (referred to as hPRL PitProKO BAC). We compared the transcriptional behavior of the 2 constructs when they were stably transfected into GH3 pituitary cell lines and found that the alternate promoters directed distinct patterns of transcriptional bursting in single cells. We examined the role of Pit-1 binding sites and the effect of modulation of Pit-1 levels in pituitary cells and found that rather than just being necessary for transcription, the binding of Pit-1 regulates the timing of hPRL transcription. These data suggest a new and unsuspected function of this well-studied transcription factor in regulating the dynamics of pituitary-specific gene expression in an important tissue model for the control of endocrine function.

## Material and Methods

### Generation of hPRL pituitary promoter knockout BAC (hPRL PitProKO BAC)

The generation of hPRL WT BAC with Luc reporter has been described previously (18). The hPRL WT BAC spans 163 kb of the human PRL genomic locus including 115 kb upstream and 38 kb downstream of the PRL gene (10 kb) and expresses the Luc reporter gene under the control of both the hPRL proximal and alternative promoters. To study specific prolactin gene activation via the alternative promoter, we sought to remove pituitary-specific promoter elements from the hPRL WT BAC by the targeting strategy based on the seamless recombineering technology (28). Briefly, the ~5kb hPRL proximal promoter was first replaced with the *GalK* (*E.coli* galactokinase k gene) coding sequence. A positive recombinant selected on galactose containing minimal media was subjected to a second round of recombination to remove the inserted GalK sequence and replace with the short (256 bp) immediate hPRL promoter sequence necessary for transcription initiation, which also contains the alternative RNA splice acceptor site at position −246 relative to Luc translation start site (18). Within this region, 3 Pit-1 binding sites are found; these elements were mutated in vitro (see following discussion of site-directed mutagenesis) prior to recombination to remove remnants of pituitary-specific regulatory elements. Positive recombinants were identified through glycerol/deoxygalactose screening and BAC size and integrity confirmed by pulse field gel electrophoresis. Mutations in Pit-1 binding sites were confirmed by sequencing. The following 2 primer pairs (italics denote homology arm sequence) were used for primary and secondary recombination respectively:

Chimeric PrlGalK-F *GAAATCGTAACTGATAAAAAATC AGCTTGACTATATCTATTGATTCTCAGACCTGTTGACAATTAATCATCGGCATAGTATATCG*.Chimeric Prl GalK-R *GTCTCACGGTTTTCTCTTTCCCAGATATTGGCTTTATAAACCTTTGATATCTTCTCAGCACTGTCCTGCTCCTTGTGA*.
Chimeric Pit1-3-F *GAAATCGTAACTGATAAAAAATCAGCTTGACTATATCTATTGATTCTCAGACTCACCTTCATCTTTCTCTC*.
Chimeric Pit1-1-R *GTCTCACGGTTTTCTCTTTCCCAGATATTGGCTTTATAAACCTTTGATATCTTCAGGTACCGAATGAATCAGGC*.

### Site-directed mutagenesis

The hPRL 5 kb Luc plasmid used for generation of the 256 bp intermediate hPRL promoter sequence has been described previously (29). Site mutations were induced in the 3 Pit-1 binding sites contained within the 256 bp region (13,30) present in the proximal pituitary promoter (−250/+1) using the QuikChange™ Site-Directed Mutagenesis Kit (Stratagene, Cambridge, UK) following the manufacturers guidelines. Oligonucleotides used for the mutation of Pit-1 sites are site 1, 5’-gcctgattcatt**CGC**t**A**c**C**tgaagatatcaag-3′, site 2, 5’-tcttcctgaatatg**G**at**CC**gaaataaaatacc-3′, and site 3, 5’-cttttggcctaat**CC**at**GG**aaatccttcctag-3′. Capital letters represent mutated bases, which resulted in the introduction of unique restriction sites as Kpn1, BamH1, and Nco1, respectively. These restriction sites were subsequently used for screening the mutated clones. The 256 bp region with mutated Pit-1 binding sites was amplified with BAC homology arms appended to primers and recombineered into the hPRL BAC Luc construct with deleted proximal promoter.

### Cell culture and generation of stably transfected BAC cell lines

Pituitary GH3 cells were maintained in phenol red-free DMEM with pyruvate/glutamine and 10% fetal bovine serum (FBS). Jurkat cells were cultured in RPMI (Life Technologies Inc., Gaithersburg, MD, USA) supplemented with glutamine and 10% FBS. Serum starving conditions were in media containing 0.25% bovine serum albumin in place of FBS.

For stable transfection of cell lines, BAC DNA (hPRL WT BAC or PitProKO BAC) was prepared by maxiprep (BAC100 Nucleobond kit, Macherey-Nagel, Germany) and 3 μg was used to transfect 10^6^ GH3 cells in a 10 cm dish or 2 × 10^6^ Jurkat cells in 25 cm^2^ flask using ExGen500 transfection reagent. Media was changed 48 h post transfection and supplemented with 500 μg/mL G418. Media and antibiotic were refreshed every 3 to 4 days. Colonies formed 2 to 3 weeks after culturing in selective media were recloned into individual wells of a 48-well plate. The stable transfectant clones that were found positive for Luc expression were sequentially scaled up to large culture vessels as necessary.

### Endpoint luminometry assays

GH3 cells (1 × 10^6^) were washed once with PBS then lysed using 200 μL of lysis buffer (25 mM Tris/PO4, 10 mM MgCl_2_, 5 mm EDTA, 15% glycerol, 0.1% Triton X-100, and 0.1 mg/mL bovine serum albumin). Cell lysis was aided by agitation at room temperature for 15 min, adenosine triphosphate added to a final concentration of 1 mM and Luc activity of samples measured using a FLUOstar Omega (BMG Labtech). Each experiment was performed in duplicate in 3 independent studies.

### Live-cell luminometry assays

Pituitary GH3 cells (1.5 × 10^4^ per well) were seeded into 96-well microplates (white opaque, PerkinElmer) in serum-free media containing 1 mM luciferin (Biosynth). After 24 h, cells were stimulated as indicated and microplates sealed with Breathe-Easy sealing film (Sigma). Luciferase activity from each well was measured using FLUOstar Omega (BMG Labtech) over a period of 24 h while maintaining the cells at 37°C in the presence of 5% CO_2_. Photon counts from each well were integrated over 5 s after every 15 min. Results are shown as mean fold induction relative to an untreated control and represent triplicates of 3 independent experiments.

### Chromatin immunoprecipitation assay

GH3 cells containing hPRL WT BAC or hPRL PitProKO BAC (3.5 × 10^6^) were seeded in 10-cm dishes and left for 48 h. Chromatin immunoprecipitation (ChIP) assay was performed as described previously (31) based on the protocol by Upstate Biotechnology. Immunoprecipitation was carried out using 5 μg of either anti-Pit-1(X-7; Santa Cruz (32)) or a nonspecific immunoglobin G (Santa Cruz). DNA was purified and amplified by polymerase chain reaction (PCR) with the following primers: (i) Pit-1 3 forward 5’-AAATCCTTCCTAGAATGTTC-3′ and Pit1 1 Reverse 5′- AATCAGGCATTCGTTTC-3′ amplifying 145 bp of DNA and (ii) rat GAPDH forward 5′- GAAATGGGCTTAGGGGTGAT-3′ and reverse 5′- TTAAGGATGGCCTTGGACTG-3′.

### Reverse transcription PCR

Total mRNA was extracted from GH3 and Jurkat cells stably transfected with PRL-Luc BAC or 5 kb Null BAC using an RNeasy kit (Qiagen, Valencia, CA, USA). Genomic DNA was removed using genomic DNA eliminating columns provided with the kit. First-strand complementary DNA was synthesized using random octamers in VILO Superscript II (Life technologies). Reverse transcription PCR for the detection of pituitary or extra-pituitary derived Luciferase mRNA was performed using forward primer 98 (7) in PRL exon-1a and reverse primer 11 (18) in the Luciferase gene.

### Real-time luminescence imaging

GH3 BAC transfectant cells and collagenase dispersed primary pituitary cells were cultured in 35-mm glass coverslip-based dishes (Greiner Bio-One, UK) in the presence of 10% FBS. Luciferin (1 mM) was added at least 10 h before the start of the imaging. Cells were transferred to a Zeiss Axiovert 200 microscope in a dark room, equipped with an incubator maintained at 37°C, 5% CO^2^ in humid atmosphere. A bright-field image was taken before and after the luminescence imaging to track the localization of cells. Luminescence images were obtained using a Fluar 10×, 0.75 NA objective. Images were captured using a photon-counting charge coupled device camera (Orca II ER, Hamamatsu Photonics, UK). Sequential images, each integrated over 10 min, were acquired using Kinetic Imaging software AQM6 (Andor, Belfast, UK). The same software was used for the analysis of the imaging data. Regions of interest were drawn around each single cell, and total photon counts for individual cell areas were obtained from each image. Mean luminescence intensity data were collected after the average instrument dark count (corrected for number of pixels being used) was subtracted from the luminescence signal. Each imaging experiment was performed at least 3 times, with a representative experiment presented in figures.

### Real-time luminescence imaging of primary bone marrow cells from hPRL-Luc transgenic rats

**Figure 1.**
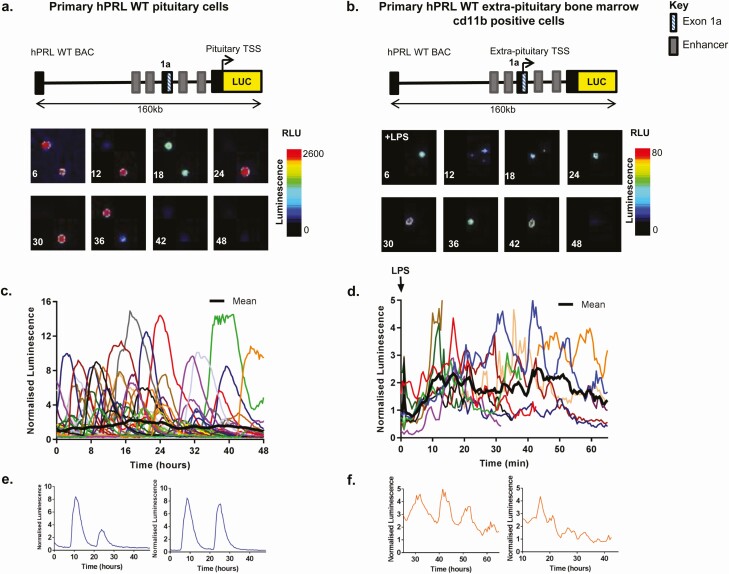
Contrasting cycles of hPRL transcription directed by the alternative and proximal promoters. Luminescence signal from primary pituitary **(A)** and bone marrow myeloid cells (following treatment with 0.05% LPS) **(B)**, taken from transgenic rats expressing luciferase under the control of a 160 kb hPRL genomic fragment (hPRL WT BAC). Colored lines represent data from single cells, **(C)** n = 45 cells and **(D)** n = 12 cells) and thick black line represents the population mean. Example single cell traces from pituitary cells **(E)** and bone marrow myeloid cells **(F)**. Luciferase activity from each cell was normalized to the average luminescence intensity of all cells in each imaging experiment at time zero.

The generation of hPRL-Luciferase rats has been described previously (18). Bone marrow cells were harvested from male hPRL-Luc rats, and erythrocytes were removed with ammonium chloride lysis solution. Bone marrow cells were cultured in RPMI 1640 containing 10% (V/V) FBS in 35-mm glass coverslip based dishes (Greiner Bio-One, UK) and left to adhere. Luciferin (1 mM (Biosynth)) was added at least 6 h before the start of the experiment. Cells were imaged with a Zeiss Axiovert 200, equipped with an XL incubator (maintained at 37°C in a 5% CO_2_ in humidified conditions) in a dark room. Luminescence images were collected using a Fluar 10×, 0.5-NA objective (Zeiss), and captured using a photon-counting charge coupled device camera (Orca II ER; Hamamatsu Photonics). Sequential images were taken with a 30-min integration period and then analyzed using Kinetic imaging software AQM6 (Andor). Lipopolysaccharide (LPS) was added directly to the dish (0.05% final concentration). Imaging experiments were performed 3 times, with a representative experiment shown in Fig. 1B.

### Pit-1 siRNA knockdown

For siRNA transfections 1.5×10^5^ hPRL WT GH3 cells were seeded into 12 well plates with 15 pmol of Pit-1 Stealth RNAi^TM^ (ThermoFisher Assay ID HSS108266) in the presence of Lipofectamine RNAimax reagent (ThermoFisher Scientific) according to the manufacturers’ protocol. Cells were also transfected in the same manner with Stealth RNAi^TM^ small interfering (siRNA) negative control, medium guanine-cytosine duplex to control for sequence independent effects. Seventy two hours after transfection cells were used for live cell luminescence imaging experiments or lysed to assess the efficiency of Pit-1 knockdown.

### Cell lysis and immunoblot analysis

After transfection with either Pit-1 siRNA, siRNA negative control or lipofectamine RNAimax reagent only, hPRL-WT GH3 cells were lysed with Laemlli sample buffer [2% SDS, 60 mM Tris-Cl (pH 6.8) 0.02% bromophenol blue, 0.1 M dithiothreitol]. Proteins were resolved by SDS-PAGE, transferred to nitrocellulose and blocked in 5% nonfat milk. Blots were incubated in primary antibodies over night at 4^o^C (Abcam), washed, and then incubated with horse radish peroxidase conjugated secondary antibodies and developed using enhanced chemiluminescence. Efficiency of Pit-1 knockdown was assessed in at least 3 experiments.

### Stochastic switch model analysis

To quantify the duration of transcriptional phases raw single-cell luminescence data from 3 independent experiments for each imaging condition (hPRL WT, PitProKO, hPRL WT Pit-1 knockdown, and hPRL WT scrambled siRNA experimental conditions) was analyzed using a previously developed stochastic switch model (SSM) (33). This model utilized a reversible jump Markov chain Monte Carlo algorithm using back-calculated transcription rates from an observed Luc signal to identify transcriptional switches, using known Luc protein and mRNA degradation rates previously reported in GH3 cell lines, as used in the present study (33). This analysis was performed using Matlab 2014a software (MathWorks), including the Bioinformatics and Statistical toolboxes.

## Results

### Contrasting cycles of hPRL transcription directed by the alternative and proximal promoters

Pulsatile cycles of prolactin transcription have previously been shown in single cells from pituitary cell lines and in primary pituitary cells (17-20,29,34-37). hPRL promoter directed reporter gene expression has also been observed in primary cultures of bone marrow myeloid cells (taken from transgenic rats expressing Luc under the control of the hPRL WT BAC), where it is driven by the alternative promoter. This expression is significantly enhanced through cytokine stimulation, such as lipopolysaccharide LPS treatment (35).

The first aim was to compare the dynamic behavior of the WT hPRL promoter (hPRL WT BAC) in pituitary and nonpituitary cells, using primary pituitary cells and bone marrow myeloid cells from hPRL WT BAC transgenic rats (Fig. 1A and 1B). Pulsatile cycles of transcriptional activity were observed with bursting characteristics in both systems (Fig. 1C and 1D). However, comparison of the observed transcription cycles suggested a shortening of the “on period” in extra-pituitary cells, in which cycles of transcription are driven by the alternative promoter (Fig. 1E and 1F).

### Generation of the proximal-promoter knockout hPRL BAC Luc (hPRL PitProKO BAC) construct

**Figure 2.**
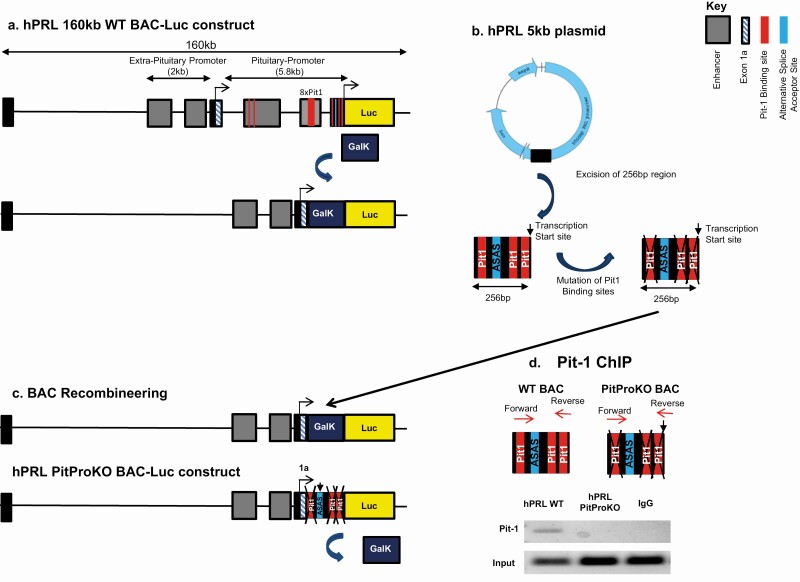
Generation of the hPRL PitProKO-Luc BAC. Schematic illustration of the knockout of the prolactin proximal pituitary promoter using a 160kb hPRL BAC-Luc construct (hPRL WT). 5 kb pituitary promoter sequence was replaced with positive selection marker Galk, via a seamless BAC recombineering strategy to knockout the PRL pituitary promoter **(A)**. Site-mutations were introduced in the 3 Pit-1 binding sites present in the proximal pituitary promoter (−256/+1) using hPRL 5 kb Luc plasmid **(B)**. 256 bp DNA fragment containing mutated Pit-1 sites along with alternative acceptor site was amplified from hPRL 5kb Luc plasmid using BAC homology arms and inserted into the pituitary promoter knockout BAC replacing the Galk gene, thereby creating a construct expressing luciferase under the control of the extra-pituitary promoter (PitProKO) **(C)**. GH3 cells containing hPRL WT or hPRL PitProKO BAC constructs were fixed in 1% formaldehyde and subjected to ChIP with either nonspecific immunoglobin G antibody or Pit-1 specific antibody. DNA was extracted and amplified by primers flanking the first and third pit-1 binding sites or a non-specific rat GAPDH sequence **(D).** Abbreviations: GalK, galactokinase; Luc, luciferase.

**Figure 3.**
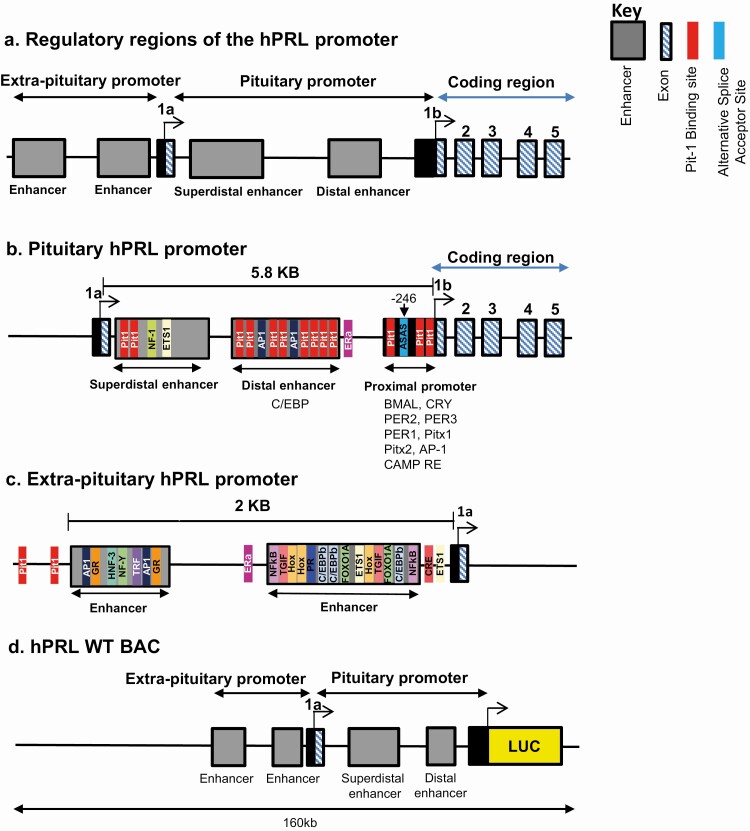
Schematic of the regulatory elements within the hPRL pituitary and extra-pituitary promoter regions. Schematic of the hPRL promoter illustrating pituitary and extra-pituitary regions **(A).** Response elements within the pituitary promoter that enable expression of PRL in the pituitary (adapted from (4)) **(B)**. Response elements within the extra-pituitary region that enable expression of PRL in extra-pituitary tissues (including information from (38)) **(C)**. Schematic of the hPRL-Luc 160 kb BAC expressing luciferase under the control of the entire hPRL gene **(D)**. Abbreviations: AP1, activator protein 1; cEBPβ, CCAAT binding protein beta; CRE, cAMP response element; ER, estrogen receptor; FOXO1A, forkhead box protein O1A; GR, glucocorticoid receptor; HNF-3, hepatocyte nuclear factor 3; NFκB, nuclear factor kappa-light-chain-enhancer of activated B cells; NF-Y, nuclear factor Y; PR, progesterone receptor; TGIF, TG-interacting factor; TRF, TBP (TATA binding protein)-related factor.

To evaluate the influence of promoter architecture on transcriptional bursting the hPRL WT BAC was modified to delete the entire 5 kb proximal pituitary promoter, leaving the upstream exon 1a and the alternative promoter intact (Fig. 2A). This provided a construct in which Luc expression was directed from the upstream alternative hPRL promoter, enabling direct comparison with the hPRL WT construct. The hPRL proximal promoter contains an alternative splice acceptor site (ASAS) at position −246 relative to its transcription start site, which is important for the function of the alternative promoter (18) (for diagrammatic analysis of the region and constructs see Fig. 3). In addition, this region contains 3 Pit-1 binding sites, which could have the potential to drive expression from the pituitary promoter. We therefore performed site-directed mutagenesis to remove the 3 Pit-1 binding sites in the hPRL Luc 5kb plasmid (described previously (29); Fig. 2B). After mutation, a 256 bp DNA sequence containing the ASAS and mutated Pit-1 binding sites was amplified from the hPRL Luc 5kb plasmid and inserted into the hPRL Luc BAC with the 5kb pituitary promoter region deleted (Fig. 2C). ChIP confirmed absence of Pit-1 binding to the mutated construct (Fig. 2D). The resulting hPRL BAC, termed PitProKO BAC, expresses Luc under the control of the alternative promoter, and contains the ASAS and mutated Pit-1 sites. The remaining putative Pit-1 binding sites in the alternative promoter have previously been found to be nonfunctional (7) and were therefore not removed or mutated.

### The hPRL alternative promoter is active in GH3 pituitary cells independently of Pit-1

**Figure 4.**
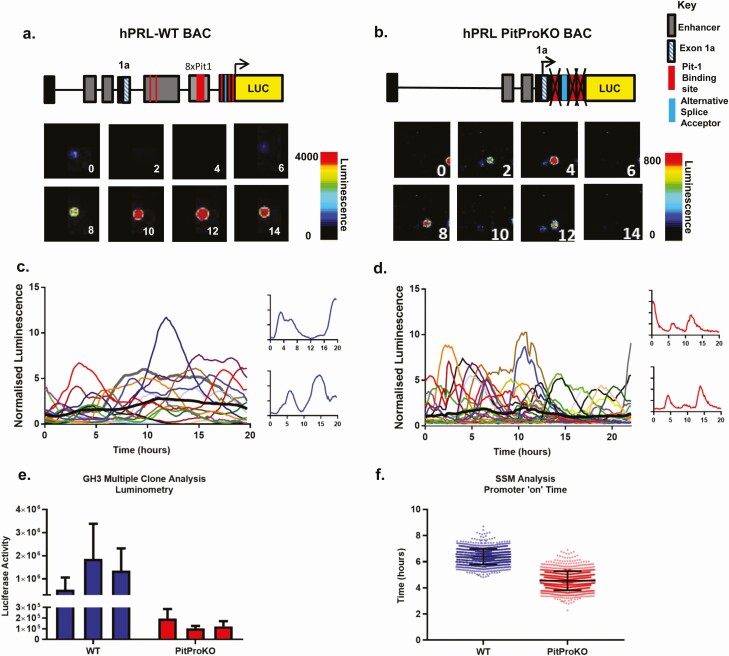
Bursting gene transcription of the hPRL alternative promoter. Single cell transcriptional activity in GH3 hPRL WT and PitProKO cell lines. Representative images and single cell transcriptional traces from hPRL WT **(A** and **C)** and hPRL PitProKO cells **(B** and **D)**. Colored lines represent data from single cells with the thick black line representing the mean response of the population; n = 16 **(C)** and n = 28 **(D)**. Luciferase activity from 3 individual hPRL WT and 3 hPRL PitProKO clonal GH3 cell lines **(E).** Luciferase activity from each cell was normalized to the average luminescence intensity of all cells in each imaging experiment at time zero. Estimated transcriptional “on” time determined by SSM compared between hPRL WT and PitProKO cells. Bars show standard deviation of the cycle length from 56 and 69 cells, respectively, in 3 experiments per cell type. The duration of an “on phase” is significantly reduced in PitProKO cells (Kolmogorov-Smirnov, *P < 0.05) **(F)**.

The hPRL alternative promoter is often referred to as the extra-pituitary promoter, based on the notion that its activity is confined to sites outside of the pituitary. Here, to assess the contribution the alternative promoter plays in the transcriptional regulation of the hPRL gene in the pituitary, we generated rat pituitary GH3 stable cell lines expressing hPRL WT BAC-Luc or hPRL PitProKO BAC-Luc. Incorporation of the entire BAC without truncation into the cell lines was confirmed following genomic DNA extraction, with PCR using primer sets spanning the entire BAC construct, including the deleted 5 kb region (Supplemental Figure S1 (39)). The Luc expression levels of 3 WT and 3 PitProKO clonal GH3 cell lines were compared. All cell lines displayed high and fluctuating levels of reporter gene activity (Fig. 4A-4E). However, WT clonal lines showed markedly higher Luc activity compared with PitProKO clones (as seen in Fig. 4A and 4B, with quantitative luminometry in Fig. 4E). These data confirm that the alternative promoter is active in pituitary cells but that the activities of both promoters are required for higher-level gene expression.

To confirm bona fide alternative promoter activity, reverse transcription PCR was performed using GH3 hPRL WT cells and a primer set to amplify from exon1a and a portion of the Luc gene. A single 423 bp product was detected that corresponded to the splicing of exon1a into exon 1b, thus bypassing the pituitary 5′UTR sequence, which contains Pit-1 binding sites (Supplemental Figure 1B (39)). A single product (670 bp) was also detected in the nonpituitary lymphoblastoid Jurkat cell line expressing the hPRL WT construct. This was 246 bp longer than its pituitary counterpart and corresponded to a splicing of exon 1a to a position −246bp upstream of transcriptional start site. These results together show that Luc expression observed from PitProKO cells is representative of hPRL alternative promoter activity and that both proximal and alternative promoters contribute towards hPRL directed Luc activity in GH3 pituitary cells.

Functional activation of the alternative promoter in GH3 PitProKO cells was confirmed by measuring the transcriptional response to appropriate stimuli. Induction was markedly greater in hPRL-WT cells where both promoters were present (Supplemental Figure S2A (39)). The PitProKO promoter was nonresponsive to both estradiol (E2) and tumor necrosis alpha treatment (Supplemental Figure S2B (39)), supporting our previous studies demonstrating that the hPRL transcriptional response to these stimuli is mediated by an estrogen response element in the proximal promoter (−1189 bp from the transcription start site), which is not present in the PitProKO construct (17,40).

### The hPRL alternative promoter displays bursting gene transcription

We have previously shown the pulsatile nature of hPRL transcription in pituitary cells using both 5 kb and BAC constructs in pituitary cell lines and in normal pituitary tissue in transgenic rats (17,20,25,27-32). The recombinant BAC constructs allowed us to test whether this bursting transcriptional behavior was specific to activity directed by the proximal promoter and whether Pit-1 binding elements were required. Real-time luminescence imaging was conducted over 20-h periods in living cells to directly compare the dynamics of Luc expression from single unstimulated hPRL WT and PitProKO cell lines (Fig. 4A-4D). Cyclical patterns of transcriptional activity were observed in both cell lines, with lower amplitude in the PitProKO cells (normalized data shown in Fig. 4C and 4D). The amplitude difference was comparable to the difference in Luc activity observed between hPRL WT and PitProKO clonal cell lines seen in Luc assays (Fig. 4E). In addition to a reduction in amplitude, analysis of single cell traces also revealed that the active “on periods” of gene transcription appeared significantly shorter in PitProKO cells than in WT cells, in other words that the pulses seemed to be shorter and sharper than in the WT cells (Fig. 4C and 4D insets). To quantify the differences in transcription timing between the 2 promoters, single-cell luminescence data were analyzed using a previously described stochastic switch model (33). This modeling analysis confirmed a significant reduction in the “on” time of the PitProKO promoter compared to WT cells (Fig. 4F), confirming the initial impression that the peaks of transcriptional activity were briefer. These data show that the hPRL alternative and proximal promoters both generate cyclical patterns of transcription, but that transcription in the absence of Pit-1 binding occurs at a reduced rate, with significantly shorter periods of active transcription in any given cycle.

### Binding of the pituitary-specific transcription factor Pit-1 to cis-acting regulatory elements plays a key role in prolonging the duration of “on periods” of active gene transcription

**Figure 5.**
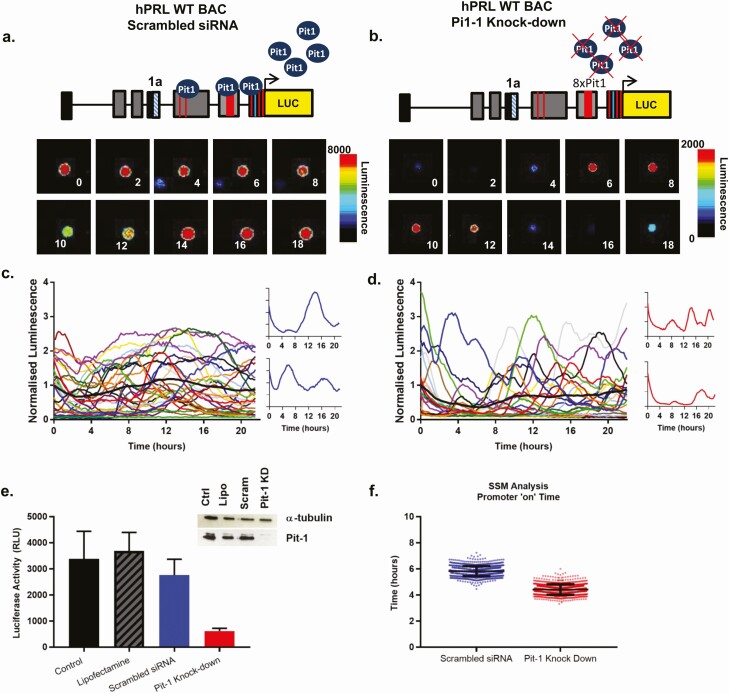
Pit-1 binding regulates the timing of hPRL transcription. Single-cell transcriptional activity of hPRL WT cells following siRNA Pit-1 knockdown or siRNA negative control, 72 h after transfection. Representative images and single-cell transcriptional traces from siRNA negative control treated hPRL WT **(A** and **C)** and hPRL WT, Pit-1 siRNA knockdown cells **(B** and **D)**. Colored lines represent data from single cells with the thick black line representing the mean response of the population; n = 30 cells **(c)**, n = 22 cells **(D)**. Luciferase activity from each cell was normalized to the average luminescence intensity of all cells in each imaging experiment at time zero. Luciferase activity **(E)** and Pit-1 protein expression **(E inset)** of hPRL WT cells following treatment with lipofectamine transfection reagent, siRNA negative control or Pit-1 siRNA knockdown (KD). Estimated transcriptional “on” time determined by SSM, compared between hPRL WT scrambled siRNA, and hPRL WT siRNA Pit-1 knockdown. Bars show SD of the cycle length from 77 and 62 cells, respectively, in 3 experiments per cell type. The duration of an “on phase” is significantly reduced in cells with Pit-1 knockdown (Kolmogorov-Smirnov, *P < 0.05) **(F)**.

One major factor that could explain these differences in transcription timing was the presence or absence of Pit-1 binding to the different hPRL promoters, in different cell contexts. We hypothesized that rather than simply being required for pituitary hPRL gene expression, Pit-1 might also play a role in the timing of transcription and contribute to the stabilization of efficient and productive transcription cycles. To test this possibility, siRNA was used to knock down Pit-1 expression in both cell lines, comparing the behavior of the WT and PitProKO constructs (Fig. 5).

Knockdown of Pit-1 reduced transcriptional activity of the WT BAC construct, as seen in luminometry assays (Fig. 5E), where Pit-1 expression was successfully reduced by greater than 95% (Fig. 5E inset). Quantitative analysis of single-cell transcriptional data by SSM confirmed that knockdown of Pit-1 resulted in a significant shortening of the on-period of transcription (Fig. 5F), consistent with the findings from the BAC mutagenesis studies. The PitProKO BAC contains mutated Pit-1 binding sites; therefore, knockdown of Pit-1 in these cells should have no effect on the timing of reporter gene expression. SSM analysis of single-cell imaging data showed no difference in the “on periods” of active transcription between PitProKO cells and in PitProKO cells in which Pit-1 expression was successfully knocked down (Supplemental Figure 3 (39)).

### Asymmetry between activation and deactivation during a transcriptional pulse

**Figure 6.**
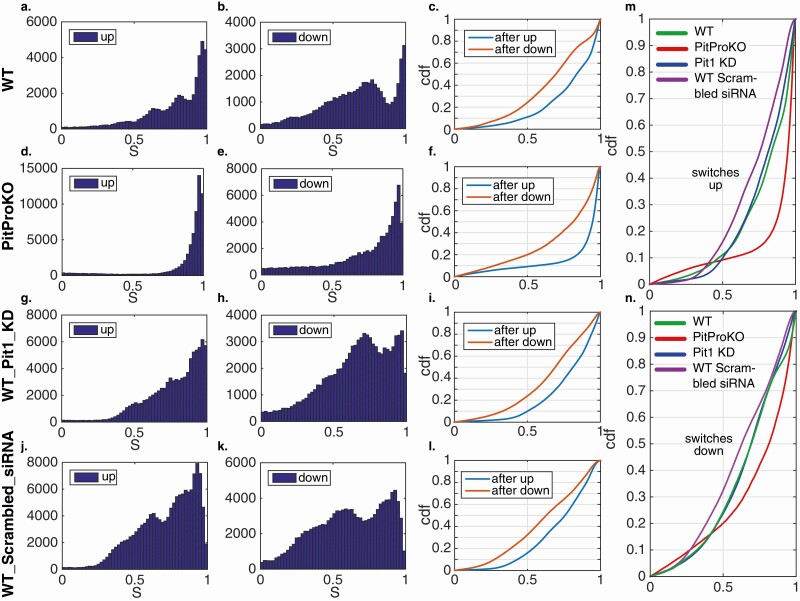
Distribution of the switching score S for up and down switching for each of WT, PitProKO, WTPit-1 knockdown, and WT scrambled siRNA. **(A, D, G, J)** The distributions of scores S for up switches for each of the 4 data sets. **(B, E, H, K)** As before (A, D, G, J) but for down switches. **(C, F, I, L)** Cumulative distribution functions for each of the 4 data sets. In each case there is a significant difference between the up and down switches, with up switches having larger values of S. (**M, N**) Comparison of the cumulative distribution functions of the 4 data sets.

In previous work (21) on the transcription of human growth hormone, we identified an asymmetry in the number and size of rate-increasing and rate-decreasing switches, resulting in a predominance of an all-or-nothing activation step, followed by a multistep graded reduction. We therefore investigated if there was such an asymmetry in the current system. To do this, each data set (WT, PitProKO, WT-Pit-1 knockdown, and WT scrambled siRNA) was processed by the SSM and to each switch in the transcription rate we associated a score S, which is the ratio of the change in the rate to the highest of the rates before and after the switch. Thus, a switch with S close to 1 is more binary (complete) while one with a smaller S is partial. We found that for each construct and condition considered, the distributions of S for up and down switches were significantly different with the asymmetry clearly present. The down switches had substantially more partial events than the up switches, which were dominated by complete switches (Fig. 6). The distributions found for the PitProKO cells substantially differed from the other constructs. For both the up and down switches, there was a highly significant greater tendency for complete transitions and a binary response (Fig. 6M and 6N) when just the alternative promoter was available. This may be partly explained by the fact that the switches are smaller in magnitude. On the other hand, the distributions for the hPRL WT cells and those with the Pit-1 knockdown were very similar as can be seen from the cumulative distribution functions in Fig. 6M and 6N. This suggests that the proximal pituitary promoter is responsible for the more graded response seen in the WT but that this response is not due to interaction of Pit-1 with the promoter. We hypothesize that the graded response is associated with extra transcriptional availability modulated by the proximal pituitary promoter and that Pit-1 binding facilitates higher transcription rates during the pulses.

## Discussion

Heterogeneity in gene expression within a cell population has been observed in numerous single-cell imaging studies where it has been shown in a diverse range of organisms that individual genes can be transcribed in short bursts of variable duration and frequency (19,22-26,37,41). Evidence supports several molecular mechanisms that together control and modulate transcriptional bursting, including nucleosome occupancy (42), chromatin modifications (19,23,43), transcription factor availability (23,26,43,44), and promoter structure (23) (reviewed in (45)). We have used pituitary cells as a model system and studied the transcriptional bursting from two alternative promoters in the hPRL gene, in the presence or absence of the pituitary-specific transcription factor Pit-1. Using live-cell imaging of stably transfected cells, we identified the role of prolactin promoter elements in the determination of the transcriptional timing characteristics in single pituitary cells. We find kinetically distinct transcriptional bursting behavior of the 2 alternative promoters within the prolactin gene and that binding of Pit-1 to prolactin promoter elements prolongs the duration of “on periods” of active gene transcription.

We previously generated a BAC reporter construct (hPRL WT) spanning 163 Kb of the human PRL genomic locus and engineered it to insert the Luc reporter gene at the start of the hPRL exon 1b, thus preventing expression of the hPRL coding sequence (18). We have studied the behavior of this hPRL WT BAC reporter construct using in vivo and in vitro models (18,20,34,35). Whole body in vivo imaging of hPRL-Luc BAC-transgenic rats using this construct revealed striking evidence of alternative promoter activation after immune challenge, demonstrating that this construct can display transcriptional control by both the exon 1b promoter and the alternative upstream exon 1a promoter (20,35). To determine the relative contributions of the 2 alternative promoter regions, in the present study we engineered the hPRL-Luc BAC (referred to as the WT construct) by deleting the entire 5 kbp region of proximal promoter and inserting a 256 bp DNA fragment which contained alternative splice acceptor site and mutated nonfunctional Pit-1 binding sites (hPRL PitProKO BAC). This strategy resulted in a functional upstream exon 1a promoter driving the Luc reporter gene and hPRL gene exons and introns, together with 115 kb upstream and 38 kb downstream flanking regions but no functional Pit-1 responsive elements. The initial assumption in this work was that the two promoters in the hPRL gene locus would display clear differential cell-type-specific activation, namely, that the exon 1b promoter with its multiple Pit-1 binding sites would generate “pituitary-specific” activation and that the upstream exon 1a promoter, which lacks Pit-1 binding sites, would be active only in nonpituitary cells (10). The recombinant BAC approach also allowed us to evaluate possible differences in transcriptional timing in relation to promoter structure and transcription factor binding.

Alternative promoters are a common occurrence in the mammalian genome and can allow diversity and flexibility in gene expression (46,47). The hPRL proximal and distal promoter regions differ greatly in their architecture with distinct enhancer and regulatory region configuration (Supplemental Figure 1 (39)). A major difference is the presence of multiple Pit-1 binding sites in the proximal promoter region. We have shown here that the alternative “nonpituitary” promoter is transcriptionally active in pituitary cells, albeit at a greatly reduced level. This suggests that the 2 hPRL gene promoters display tissue preference rather than absolute tissue specificity in activation. This phenomenon of tissue preference rather than tissue exclusivity has been reported in a variety of human genes (46). Single-cell analysis of reporter gene expression confirmed that both promoters displayed heterogeneous and bursting transcriptional activity, with the alternative promoter associated with a significantly reduced transcriptional “on” time and a more binary response. The short “on” timing appears to be a specific feature of alternative “nonpituitary” promoter activity as similar timing characteristics were observed in primary bone marrow myeloid cells from hPRL-WT transgenic rats (Fig. 1).

The activity of Pit-1 is regulated in response to signal-transduction pathways by its interaction with co-repressor and activator complexes containing nuclear receptor co-repressor 1 and 3′,5′-cyclic adenosine 5′-monophosphate-response element binding protein, respectively (48). In response to physiological stimuli Pit-1, through its interaction with activator complexes uses the histone acetyltransferase function of ′,5′-cyclic adenosine 5′-monophosphate-response element binding protein (48). Pit-1 has been reported to direct changes in the chromatin structure of the hPRL promoter (49), and changes in chromatin structure have been implicated in the timing of hPRL gene transcription (19). In this work we examined the effect of modifications of promoter structure by inactivation of Pit-1 binding sites, as well as reduction of transcription factor availability in Pit-1 knockdown experiments. The evidence presented here shows that burst timing is affected by the number of transcription factor binding sites and transcription factor availability. Binding of transcription factors to regulatory regions can influence burst frequency (27) and burst size (43,50) with differences in function possibly specific to certain transcription factors. Previously we have shown that pulsatile patterns of hPRL gene expression change during development; nascent primary pituitary lactotroph cells (embryonic day 16.5, from transgenic reporter rats) show “short” bursts of transcriptional activity, whereas later in development and in adult pituitary tissue a more stable transcription phenotype is observed (20,34). Pit-1 mRNA is first detected in the rat anterior pituitary at embryonic day 15 and the protein is initially expressed at very low levels (51). Our data suggest that the difference seen in transcriptional bursts during development could be due to the availability of the Pit-1 transcription factor.

An important finding of the present work was that manipulation of the promoter structure had clear effects on the timing and structure of transcriptional cycles in living, intact cells. We and others have previously found that the prolactin gene, like other genes studied, displays cycles of transcriptional activity that are likely to involve chromatin remodeling (19,23,37,52). The question arises as to what elements of promoter structure may control the existence or timing of these cycles. Pit-1 is a well-studied transcription factor thought to be necessary for tissue-specific expression of the prolactin gene in the pituitary. The prolactin gene proximal promoter contains multiple Pit-1 binding sites that are thought to mediate responses to signaling stimuli (4). Deletion of the Pit-1-regulated promoter elements in the human prolactin gene locus did not prevent transcriptional cycles from occurring but did markedly reduce the duration of active periods of transcription, modeled here as “on periods” using a stochastic switch model and also resulted in a less graded, more binary, response profile. This suggests that Pit-1 binding in the “pituitary-specific” promoter is important to stabilize transcriptional complexes for longer periods to allow higher rates of transcription to occur, and our data are the first to indicate that the action of this transcription factor has an important effect on timing.

An important challenge is to assess the physiological significance of pulsatile transcription and the role that its modulation may play on normal physiology. The present studies were performed in the pituitary GH3 cell line, which has significant limitations: it is an immortalized clonal cell line, and the cells lack functional dopamine receptors. Nonetheless, they have proved a valuable test-bed in which to explore how pituitary hormones may be regulated, and a system in which complex genetic manipulations may be trialed before conducting studies in living animals. In our previous work using transgenic hPRL-EGFP reporter rats, we found identical transcriptional pulses in living intact normal pituitary cells (18,20,34,37). In addition to studying the effects of tissue structure and development on dynamic transcriptional patterns, we showed how modification of pulse characteristics in individual cells changes the overall mRNA production in a larger population of cells (36). Important questions still to be addressed in vivo include studies on the effects of estrogen and dopamine and also how hormone production by clonal pituitary tumors might differ from that of lactotrophs in the intermingled cell populations of the normal pituitary. This work will require further use of such animal models, but in the meantime our cell line data indicate that transcriptional timing is an important aspect of overall physiological control of pituitary hormone production and that transcription factors such as Pit-1 appear to have a key role in stabilizing transcriptional pulses to ensure high-level hormone production.

In summary, Pit-1 plays an important role in the timing of transcription cycles, rather than simply being necessary to permit tissue-specific gene expression. The proximal promoter displays a binary (“all-or-nothing”) activation step, with the presence of Pit-1 and Pit-1 binding sites associated with prolongation of the subsequent “on-phase,” and multistep graded inactivation. In the absence of Pit-1 or Pit-1 binding, as occurs with the alternative upstream promoter in nonpituitary tissues, both the activation and inactivation steps of the transcriptional cycles have binary characteristics of a smaller amplitude. Pit-1 is localized in nuclear foci and dynamically partitioned, with a key role in interacting with the nuclear matrix (53). In addition, Pit-1 has been shown to reorganize long range looping (54), nuclear co-repressors (55), nucleosome location, and histone acetylation (49). The current results suggest a dynamic rather than passive role for Pit-1 in transcriptional regulation. These results may be applicable to the mechanism of action of other master cell-lineage specific transcription factors and to the dynamic behavior of genes which are differentially expressed through alternative promoters.

## Additional Information

***Data Availability:*** Some or all data sets generated during and/or analyzed during the current study are not publicly available but are available from the corresponding author on reasonable request.
